# Safety and Efficacy of a MEURI Program for the Use of High Dose Ivermectin in COVID-19 Patients

**DOI:** 10.3389/fpubh.2022.813378

**Published:** 2022-02-22

**Authors:** Marcos Alejandro Mayer, Alejandro Krolewiecki, Alejandro Ferrero, Marcelo Bocchio, Juan Barbero, Marcos Miguel, Ariel Paladini, Carlos Delgado, Juan Ramón Ojeda, Claudia Elorza, Ana Bertone, Pedro Emanuel Fleitas, Gustavo Vera, Mario Rubén Kohan

**Affiliations:** ^1^Ministerio de Salud de la Provincia de La Pampa, Santa Rosa, Argentina; ^2^Fundación Centro de Salud e Investigaciones Médicas, Santa Rosa, Argentina; ^3^Consejo Nacional de Investigaciones Científicas y Técnicas, Buenos Aires, Argentina; ^4^Instituto de Investigaciones de Enfermedades Tropicales, Universidad Nacional de Salta, Sede Regional Orán, Salta, Argentina

**Keywords:** ivermectin, COVID-19, mortality, ICU-admission, safety

## Abstract

**Background:**

In the absence of antiviral alternatives, interventions under research for COVID-19 might be offered following guidelines from WHO for monitored emergency use of unregistered and experimental interventions (MEURI). Ivermectin is among several drugs explored for its role against SARS-CoV-2, with a well-known safety profile but conflicting data regarding clinical utility for COVID-19. The aim of this report is to inform on the results of a MEURI Program of high-dose ivermectin in COVID-19 carried out by the Ministry of Health of the Province of La Pampa, Argentina.

**Methods:**

COVID-19 subjects, within 5 days of symptoms onset were invited to participate in the program, which consisted in the administration of ivermectin 0.6 mg/kg/day for 5 days plus standard of care. Active pharmacosurveillance was performed for 21 days, and hepatic laboratory assessments were performed in a subset of patients. Frequency of Intensive Care Unit (ICU) admission and COVID-19-related mortality of subjects in the ivermectin intention to treat group were compared with that observed in inhabitants of the same province during the same period not participating in the program.

**Results:**

From 21,232 subjects with COVID-19, 3,266 were offered and agreed to participate in the ivermectin program and 17,966 did not and were considered as controls. A total of 567 participants reported 819 adverse events (AEs); 3.13% discontinued ivermectin due to adverse events. ICU admission was significantly lower in the ivermectin group compared to controls among participants ≥40 year-old (1.2 vs. 2.0%, odds ratio 0.608; *p* = 0.024). Similarly, mortality was lower in the ivermectin group in the full group analysis (1.5 vs. 2.1%, odds ratio 0.720; *p* = 0.029), as well as in subjects ≥ 40 year- old (2.7 vs. 4.1%, odds ratio 0.655; *p* = 0.005).

**Conclusions:**

This report highlights the safety and possible efficacy of high dose ivermectin as a potentially useful intervention deserving public health-based consideration for COVID-19 patients.

## Introduction

COVID-19 constitutes a public health emergency at a global scale since its appearance in Wuhan, China, in December 2019 (1). By the end of January 2022, over 360 million cases and 5.5 million deaths have been reported worldwide (2). Vaccine rollout campaigns, which currently offer the best hopes for pandemic control, are a key targeted pharmacologic intervention for containment of disease spread and impact on the incidence of severe cases (3, 4).

Despite having an asymptomatic or mild course in most cases, COVID-19 constitutes a significant burden on health systems unprepared to cope with outbreaks requiring, among other things, massive testing capacity for rapid case detection and isolation, expansion of intensive care unit (ICU) capacity and case management guidelines for a previously unknown pathogen. This public health crisis has been, and still is, more profound in countries with weaker health systems (5).

The unprecedented progress in vaccine development has not been matched by the development of antiviral molecules, either new or repurposed, that could contribute to the treatment or prevention of COVID-19. With convalescent plasma, monoclonal antibodies, hydroxychloroquine and antiretrovirals among many molecules tested *in-vitro* and in observational and clinical trials, different treatment guidelines only agree in the use of corticosteroids, thromboprofilaxis and respiratory support in their recommendations (6, 7). In terms of antivirals, remdesivir has been incorporated in some treatment guidelines and more recently, some have incorporated newly developed antiviral drugs like ritonavir-boosted nirmatrelvir, sotrovimab, and molnupiravir as treatment alternatives for high-risk non-hospitalized patients based on the results of clinical trials (8).

Ivermectin (IVM) is an endectocide drug widely used for the treatment and control of onchocerciasis and lymphatic filariasis through mass drug administration programs, which has a wide therapeutic index and a benign safety profile in the currently approved doses of 150–400 μg/kg mostly in single dose regimens (9, 10). Besides its known uses, it has been evaluated as an antiviral, demonstrating *in vitro* activity against zika, rabies and dengue among other viruses (11). In the case of dengue, a recently published randomized clinical trial from Thailand showed positive although inconclusive results (12). For SARS-CoV-2, early on the pandemic, the report of the antiviral activity of IVM in Vero cells cultures sparked widespread interest in the potential utility of this oral, safe and affordable drug against COVID-19 (13). However, after over a year of several publications addressing this question, there is a lack of clear evidence for or against the use of IVM in COVID-19 patients (6, 14). With at least two completed double-blind randomized clinical trials (RCTs) showing no effect in clinical endpoints, other smaller randomized trials using higher doses identified significant antiviral effects (15–19) or a reduction in the clinical signs, including anosmia (20). That undefined landscape is summarized by the current NIH COVID-19 treatment Guidelines stating that there is insufficient data to recommend either for or against the use of IVM in COVID-19 patients (6).

In 2016, the World Health Organization (WHO) issued the Guidance for Managing Ethical Issues in Infectious Diseases Outbreaks, with the aim of complementing existing guidance on ethics in public health in situations of great uncertainty and including recommendations for the use of unproven interventions outside clinical trials, which based on a WHO response developed in the context of the outbreak of Ebola Virus Disease in Western Africa in 2014 are called “monitored emergency use of unregistered and experimental interventions” (MEURI) (21). These interventions apply when no proven effective treatments exist, it is not possible to initiate clinical trials immediately, existing preliminary data supports the intervention, relevant regulatory, ethical and scientific authorities approve such use, resources are available to minimize risks and patient's informed consent is obtained. Proper monitoring and timely sharing of the results with the wider medical and scientific community are also requirements to MEURI activities.

The aim of this report is to inform about the satisfactory safety and efficacy results of a MEURI Program for the use of high dose IVM in COVID-19 patients, carried out by the Ministry of Health of the Province of La Pampa, in the Patagonian region of Argentina.

## Methods

### MEURI Program for the Use of High Dose IVM in COVID-19 Patients

By the end of January 2021, the Ministry of Health of the Province of La Pampa (Argentina) authorized the implementation of a MEURI program based on the use of high dose IVM (600 μg/kg for 5 days) in COVID-19 adult patients (older than 18 year-old). In order to be able to participate, subjects had to be able to provide written informed consent, have a confirmed diagnosis of COVID-19 infection (by means of RT-PCR or antigen test) and symptoms onset within 5 days before entering the program. Exclusion criteria included pregnancy, breast feeding, hypersensitivity to IVM or acute allergic states, and active use of warfarin. Women with child-bearing potential were eligible if they were taking effective contraceptive measures before entering the program and agreed to continue with these measures for at least 30 days after receiving the last dose of IVM. Meanwhile ambulatory and inpatient subjects were allowed to participate (as long as they accomplished all inclusion criteria and had no exclusion criteria), admission to ICU was considered an exclusion criteria. Every member of the health staff of the Province of La Pampa was instructed to invite to participate in the program to every COVID-19 patient identified within the first 5 days of symptoms onset. However, participation in the program was voluntary, and physicians could decide not to include subjects in the program based on their medical criteria.

Ethical approval was obtained from the Provincial Ethics Committee of La Pampa, and participating individuals provided written informed consent. The study protocol is registered at national registry from Argentina (RENIS), with the registry number IS003403.

### Intervention

Participants were evaluated at program entry with complete medical history and a brief physical exam. At the beginning of the program, safety laboratory assessments before and at the end of treatment were mandatory. However, after a preliminary safety analysis that triggered an amendment approved by the Ethics Committee, these assessments were no longer mandatory and could be performed or not, based on medical judgment.

Patients received oral treatment with IVM for 5 consecutive days within 30 min of food ingestion, preferentially of high fat content, at ~24 h intervals. IVM 6, 9, and 18 mg tablets were used, combined to in all cases at a dose of 0.6 mg/kg/day based on baseline weight rounding to the lower full (6 mg) dose. There were no specific guidelines regarding medical management of COVID-19 infection for the participants in the IVM program, which was the same as for the rest of the population. Standard of care in the Province of La Pampa at that time included no other antivirals and the use of systemic corticosteroids and deep-vein thrombosis prophylaxis for hospitalized cases requiring oxygen supplementation to maintain oxygen saturation ≥94%; for ambulatory cases, no disease specific interventions were included in the standard of care.

### Safety Assessment

Active pharmacosurveillance was performed during the first 21 days after treatment start by means of the completion of a follow up chart, and safety assessment was based in all subjects that participated in the program in which follow up safety data was reported.

### Hepatic Safety Assessment

Hepatic safety assessment was based on the analysis of hepatic lab exams performed before and after IVM treatment in a subset of patients, and consisted of laboratory determinations of alanine aminotransferase (ALT), aspartate aminotransferase (AST), alkaline phosphatase (ALP), and total bilirubin levels. Drug induced liver injury was defined according to the Latin American Association for Study of the liver definition, that includes (i) ALT elevation ≥5 ULN, (ii) ALP elevation ≥2 ULN (in the absence of known bone pathology driving the increase in ALP level), or (iii) ALT ≥3 ULN and simultaneous elevation of total bilirubin concentration above 2 ULN (22).

### Efficacy Analysis

In order to estimate the efficacy of the implementation of the program, the clinical evolution of the subjects in the IVM intention to treat (ITT) group was compared with that observed in inhabitants of the same province during the same analyzed period (from January 20, 2021 to May 20 2021) who did not participate in the program (control group, C). To identify them, the analysis of the National Health Surveillance System (SNVS 2.0) was used, which records, among other events, the notification of COVID-19 cases, their clinical and demographic characteristics and the respective laboratory studies, in a mandatory, nominal and immediate way, according to a national regulation.

Given that the registration methodology differs between the one used in the IVM-monitored intervention program and the one used for registering subjects and events in SNVS 2.0 database, it was decided to consider variables not dependent on the registration method in the system for the efficacy analysis. Specifically, the primary objectives of the evaluation were the analysis of the impact of the program on the frequency of ICU admission and COVID-19-related death. It should be noted that both ICU admission and death registration is carried out centrally, so their identification is independent of the type of follow-up carried out.

In order to compare the clinical course of both groups, subjects under 18 years of age and pregnant women were excluded from the analysis. Also, as part of a bias control strategy, a sub-analysis was performed after excluding subjects with neoplastic diseases.

In the univariate efficacy analysis, subgroup analysis was performed according to age group (whole sample, subjects between 18 and 40 year old or subjects ≥ 40 year old), immunization status (excluding subjects with at least one vaccine dose) and mean IVM prescribed dose.

### Statistical Analysis

Baseline characteristics of the two groups (C and IVM) were compared by means of Student's *T*-test and Chi square. The clinical evolution was evaluated by Chi square Test and logistic regression analysis. Whenever possible, number needed to treat (NNT) values for IVM were estimated for the end points of ICU admission and death. The NNT values were estimated as the inverse of the difference in estimated absolute risk between control and IVM groups. In all cases, *p*-values < 0.05 were considered statistically significant. All analysis were performed with GraphPad Prism version 9.1.0 for Windows (La Jolla, California).

## Results

### Recruitment

A total of 21,232 non-pregnant adults were identified as COVID-19 positive between January 20 2021 and May 20 2021. Of these, 3,266 agreed to participate in the program and received at least one dose of IVM, and were included in the ITT analysis group. A group of 17,966 subjects that did not participate in the program were included in the C group. Descriptive characteristics of the population are presented in Table 1.

**Table 1:** Descriptive characteristics of the sample.

**Variable**	**Control group**			**Ivermectin group**		
	**Total sample (*n* = 17,966)**	**18–40 year old subjects (*n* = 8,944)**	**Subjects ≥40 year old (*n* = 9,022)**	**Total sample (*n* = 3,266)**	**18–40 year old subjects (*n* = 1,415)**	**Subjects ≥40 year old (*n* = 1,851)**
Age (years ± SD)	42.3 ± 16.6	28.6 ± 6.1	55.8 ± 12.1	43.8 ± 15.4^**^	29.8 ± 5.9^**^	54.4 ± 11.5^**^
Sex	52.8% Female	52.8% Female	52.9% Female	50.6% Female^#^	50.1% Female	47.18% Female
	47.2% Male	47.2% Male	47.1% Male	49.4% Male	49.9% Male	48.9% Male
Complete immunization	323 (1.8%)	84 (0.9%)	239 (2.6%)	59 (1.8%)	22 (1.6%)^*^	37 (2.0%)
Incomplete immunization	1,417 (8.0%)	97 (1.1%)	1,320 (15%)	271 (8.5%)	13 (0.9%)	258 (14.2%)
Cardiovascular disease	1,792 (10.0%)	170 (1.9%)	1,622 (18.0%)	344 (10.5%)	29 (2.0%)	315 (17.0%)
COPD	1,177 (6.6%)	384 (4.3%)	793 (8.8%	244 (7.5%)	73 (5.2%)	171 (9.2%)
Hypertension	1,583 (8.8%)	67 (0.7%)	1,516 (16.8%)	551 (16.9%)^**^	27 (1.9%)^**^	524 (28.3%)^**^
Diabetes	1,579 (8.8%)	209 (2.3%)	1,370 (15.2%)	315 (9.6%)	52 (3.7%)^**^	263 (14.2%)
Neoplasm	269 (1.5%)	28 (0.3%	241 (2.7%	63 (1.9%)	10 (0.7%)^#^	53 (2.9%)
Obesity	2,241 (12.5%)	760 (8.5%)	1,481 (16.4%)	1,182 (36.2%)^**^	401 (28.3%)^**^	781 (42.2%)^**^

*COPD, Chronic obstructive pulmonary disease.*

*Complete immunization: subjects with complete vaccine scheme at least 14 days before symptoms onset.*

*Incomplete immunization: subjects with the first vaccine dose (of a two-dose scheme) received at least 14 days before symptoms onset.*

# *p < 0.05 vs. control group.*

* *p < 0.01 vs. control group.*

** *p < 0.001 vs. control group*.

**Figure 1:**
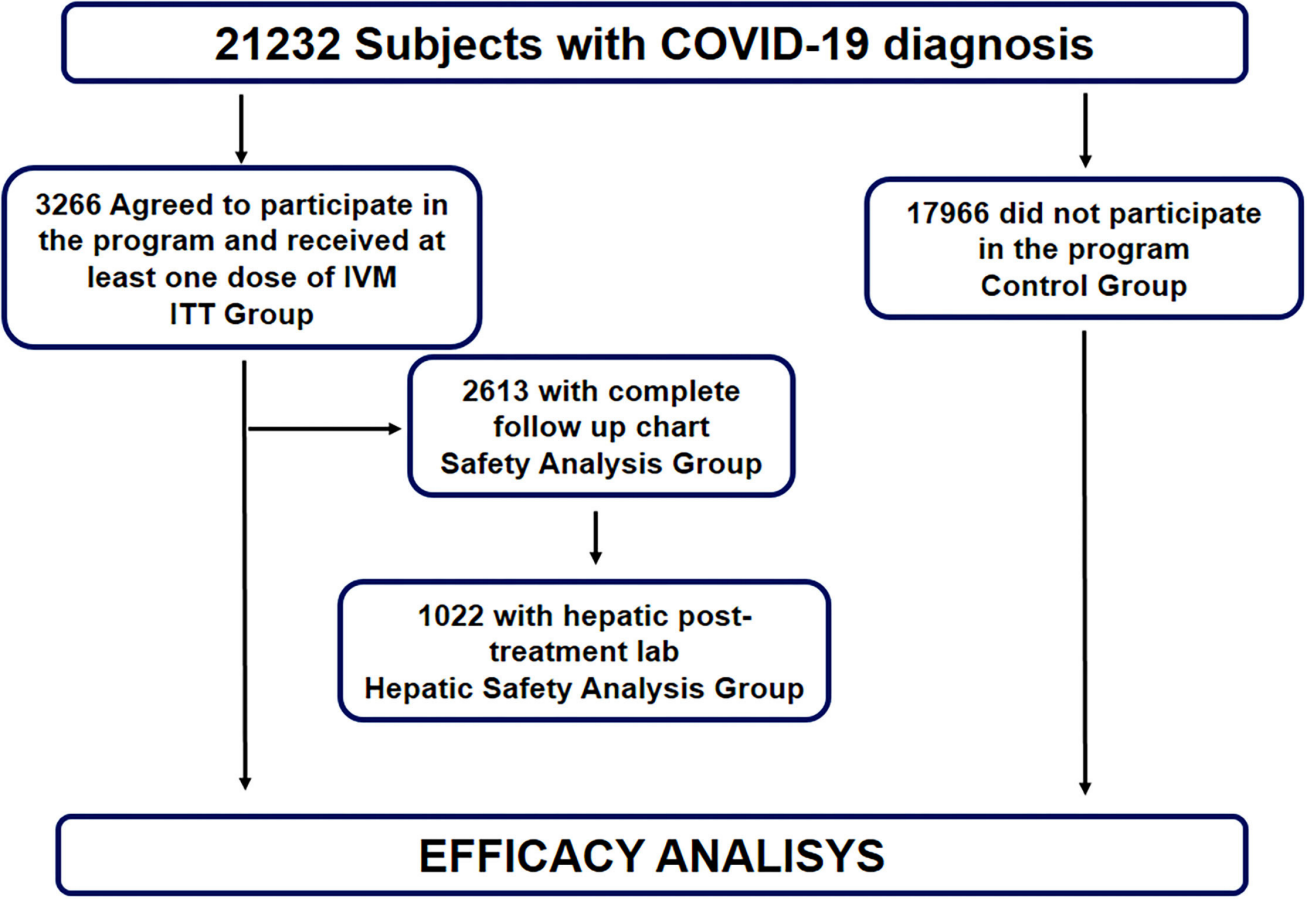
Flow diagram of the MEURI program for the use of high dose IVM in ambulatory COVID-19 patients.

Safety follow up data was obtained from 2,613 subjects that participated in the program and were included in the Safety Analysis Group. Of these, 1,022 were followed with post-treatment hepatic lab exams, and were included in the Hepatic Safety Analysis group (Figure 1).

### Safety Analysis

A total of 2,613 participants were included in the safety assessment. Five hundred sixty seven (567) participants reported 819 adverse events (AEs). Eighty-two subjects (3.13 %) discontinued IVM due to adverse events and all AEs resolved after treatment discontinuation. The most common AEs were diarrhea, followed by visual disorders, dizziness, abdominal pain, headache and nausea (Table 2). Although many of the symptoms presented during treatment resemble COVID, they were all assumed to be related to IVM treatment.

**Table 2:** Safety analysis among subjects receiving ivermectin (*n* 2,613).

**Adverse event**	***N* (%)**
Diarrhea	155 (5.93%)
Visual disorders	136 (5.2%)
Dizziness	120 (4.59%)
Abdominal pain	91 (3.48%)
Headache	73 (2.79%)
Nausea	78 (2.98%)
Anorexia	31 (1.19%)
Vomiting	30 (1.15%)
Heart rate elevation	18 (0.69%)
Rash	16 (0.61%)
Blood pressure elevation	15 (0.57%)
Pruritus	14 (0.53%)
Insomnia	14 (0.53%)
Drowsiness	14 (0.53%)

*The table shows adverse events reported in more than 0.5% of subjects*.

### Hepatic Safety Analysis

Although there was a small but statistically significant increase in ALT, AST and total bilirubin values after IVM treatment (Table 3), among 1,022 subjects that were followed up with laboratory determinations after IVM treatment, only one presented liver enzyme values compatible with low grade drug induced liver injury, that lead to drug discontinuation on day 4 of treatment. According to medical records, this subject had abnormal baseline ALT and AST values (AST 240 U/l, ALT 375 U/l, Total Bilirrubin 0.63 mg/dl and the values peaked to AST 366 U/l ALT 630 U/l total bilirrubin 0.8 mg/dl and returned to AST 111 U/l, ALT 214 U/l and total bilirrubin 0.62 mg/dl). Considering the total hepatic safety sample (*n* = 1,022), this represents an incidence of 0.98/1,000 treated subjects.

**Table 3:** Hepatic safety analysis.

**Variable**	**Pre-treatment**	**Post-treatment**
AST (*n* = 1,000)	34.07 ± 29.59 U/l	36.15 ± 31.35 U/l^**^
ALT (*n* = 988)	25.67 ± 14.93 U/l	27.61 ± 25.07 U/l^**^
Total bilirrubin (*N* = 966)	0.44 ± 0.24 mg/dl	0.47 ± 0.24 mg/dl^**^
Alkaline phosphatase (*n* = 1,000)	146.32 ± 77.27 U/l	120.60 ± 93.33 U/l^**^

** *p < 0.001 vs. pre-treatment values*.

### Program's Efficacy

In order to evaluate the program's efficacy, the clinical evolution of subjects in the IVM-ITT analysis group (*n* = 3,266) was compared with 17,966 subjects that did not participate in the program (C group).

**Figure 2:**
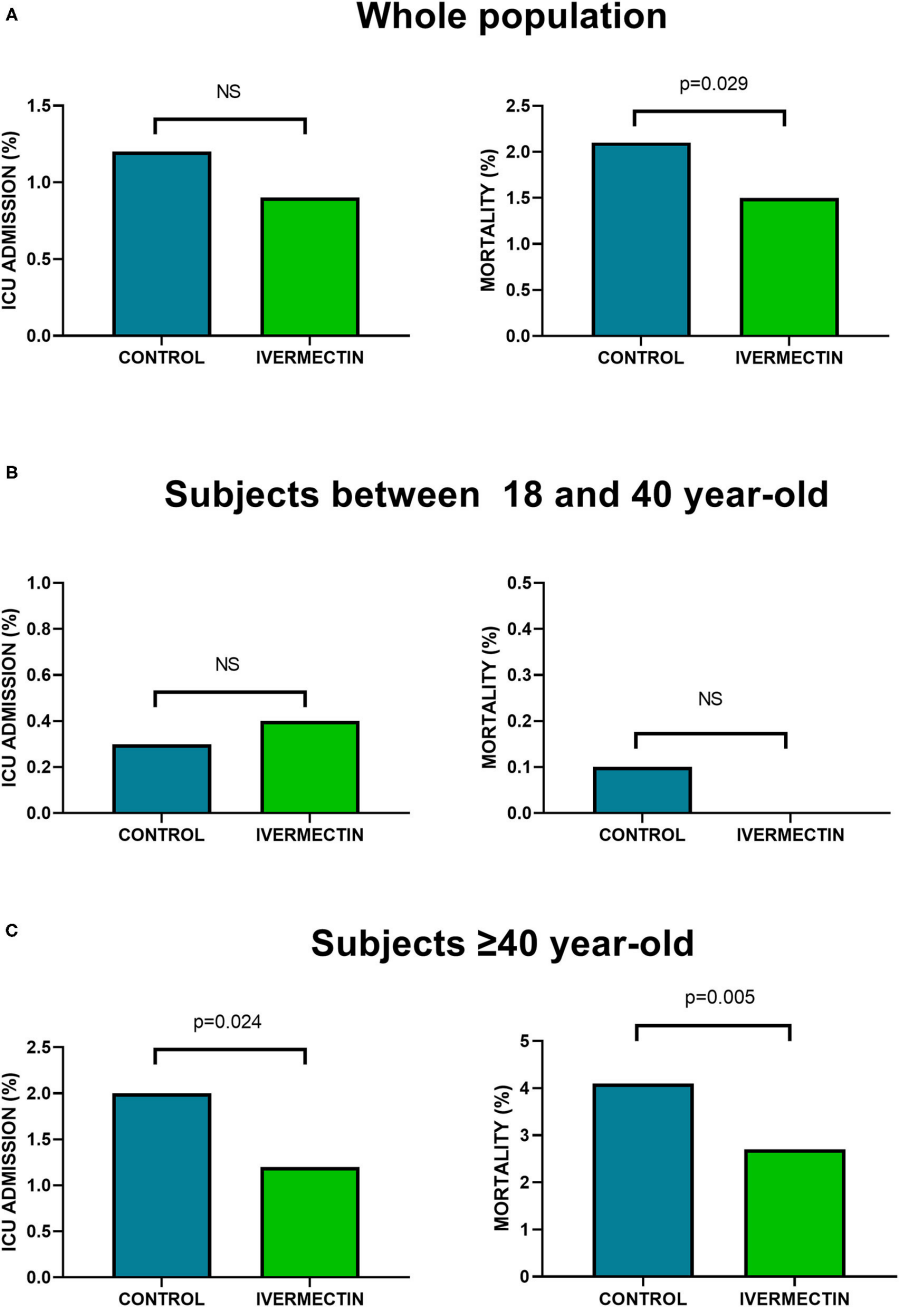
ICU admission and mortality in IVM-ITT and C groups. **(A)** Whole population: analysis of all subjects (C *n* = 17,966; IVM-ITT *n* = 3,266); **(B)** Analysis of subjects ≥18 year-old and <40 year-old (C *n* = 8,944; IVM-ITT *n* = 1,415); **(C)** Analysis of subjects ≥40 year-old (C *n* = 9,022; IVM-ITT *n* = 1,851). NS, non statistically significant; n, number of subjects.

In the whole sample analysis, there was a non-significant tendency toward lower ICU admission in the IVM-ITT group (28/3,266) compared with C (208/17,966) (0.9 vs. 1.2%, odds ratio 0.738) (95% CI 0.497–1.097, NNT 333, NS). Mortality rate was significantly lower in the IVM-ITT group (50/3,266) compared with C (380/17,966) (1.5 vs. 2.1% with an odds ratio of 0.720) (95% CI 0.535–0.969, NNT 172; *p* = 0.029) (Figure 2A).

No significant differences were observed in ICU admission (0.4 vs. 0.3%, odds ratio 1.265) (95% CI 0.484–3.310, NS) between IVM-ITT group (5/1,415) compared with C (25/8,944) in subjects younger than 40 year old. Similarly, no significant differences were observed in mortality rate in this age group (IVM-ITT group: 0/1,415 vs. C: 13/8,944) (0.0 vs. 0.1%, NS) (Figure 2B). Conversely, regarding clinical evolution of subjects ≥ 40 year old (C *n* = 9,022; IVM-ITT *n* = 1,851), ICU admission was significantly lower in the IVM-ITT group (23/1,851) compared with C (183/9,022) (1.2 vs. 2.0%, with an odds ratio of 0.608) (95% CI 0.393–0.940, NNT 128; *p* = 0.024). Mortality rate was significantly lower in the IVM-ITT group (50/1,851) compared with C (367/9,022) (2.7 vs. 4.1%, with an odds ratio of 0.655) (95% CI 0.485–0.884, NNT 74, *p* = 0.005) (Figure 2C).

Similar results were observed in the sub-analysis performed after excluding subjects with neoplastic diseases. Specifically, there was a non-significant tendency toward lower ICU admission in the IVM-ITT group (27/3,203) compared with C (202/17,697) (0.8 vs. 1.1%, odds ratio 0.736) (95% CI 0.492–1.102, NS) and a lower mortality rate in the IVM-ITT group (48/3,203) compared with C (351/17,697) (1.5 vs. 2.0%) with an odds ratio of 0.752 (95% CI 0.555–1.019) (*p* = 0.065) in the whole sample analysis. Regarding clinical evolution of subjects ≥ 40 year old, ICU admission remained significantly lower in the IVM-ITT group (22/1,798) compared with C (177/8,781) (1.2 vs. 2.0%, with an odds ratio of 0.602) (95% CI 0.385–0.941) (*p* = 0.024) after excluding subjects with neoplastic diseases, and mortality rate was significantly lower in the IVM-ITT group (48/1,798) compared with C (338/8,443) (2.7 vs. 3.8%, with an odds ratio of 0.685) (95% CI 0.504–0.931) (*p* = 0.015). No significant differences were observed in ICU admission (0.4 vs. 0.3%, odds ratio 1.270) (95% CI 0.485–3.323, NS) between IVM-ITT group (5/1,405) compared with C (25/8,916) in subjects younger than 40 year old). Similarly, no significant differences were observed in mortality rate in this age group (IVM-ITT group: 0/1,415 vs. C: 13/8,916) (0.0 vs. 0.1%, NS).

In the analysis of all non-immunized subjects (C *n* = 16,226; IVM: *n* = 2,936), there was a non-significant tendency toward lower ICU admission in the IVM-ITT group (21/2,936) compared with C (181/16,226) (0.7 vs. 1.1%, odds ratio 0.639) (95% CI 0.406–1.005, NNT 250; *p* = 0.051). Mortality rate was significantly lower in the IVM-ITT group (32/2,936) compared with C (280/16,226) (1.1 vs. 1.7%, with an odds ratio of 0.628) (95% CI 0.434–0.907, NNT 158; *p* = 0.012).

No significant differences were observed in ICU admission (0.4 vs. 0.3%, odds ratio 1.271) (95% CI 0.486–3.326, NS) between IVM-ITT group (5/1,380) compared with C (25/8,763) in non-immunized subjects younger than 40 year old, Similarly, no significant differences were observed in mortality rate between non-immunized subjects younger than 40 year old (IVM-ITT group: 0/1,380 vs. C: 13/8,763) (0.0 vs. 0.1%, NS).

Conversely, regarding clinical evolution of non-immunized subjects ≥ 40 year old (C *n* = 7,463 and IVM-ITT *n* = 1,556), ICU admission was significantly lower in the IVM-ITT group (16/1,556) compared with C (156/7,463) (1.0 vs. 2.1%, with an odds ratio of 0.487) (95% CI 0.290–0.816, NNT 95; *p* = 0.005). Mortality rate was significantly lower in the IVM-ITT group (32/1,556) compared with C (267/7,463) (2.1 vs. 3.6%), with an odds ratio of 0.566 (95% CI 0.391–0.820, NNT 66; *p* = 0.002).

A total of 2,895 subjects in the IVM group had complete data regarding weight-based ivermectin dose information (of 3,266 subjects assigned to IVM, 146 had missing data regarding the prescribed dose, and 335 had missing body weight data).

Mean ± SD IVM prescribed dose was 44.15 ± 11.83 mg/day, mean IVM prescribed dose per kg of body weight was 0.54 ± 0.09 mg/kg/day. Based on these values, two IVM groups were created: Low-dose IVM (with prescribed dose lower than 0.54 mg/kg/day, *n* = 1,157) and High-dose IVM (with a prescribed dose equal or higher than 0.54 mg/kg/day, *n* = 1,738). No significant differences were observed between groups regarding descriptive characteristics (age, gender, immunization status and comorbidities; data not shown). As expected, IVM prescribed dose per kg of body weight was significantly higher in the High-dose IVM group (mean ± SD: 0.597 ± 0.067 mg/kg/day) compared to the Low-dose IVM group (mean ± SD: 0.459 ± 0.079 mg/kg/day) (*p* < 0.001).

In the whole sample analysis of High-dose IVM vs. Low-dose IVM, there was a non-significant tendency toward lower ICU admission in the High-dose IVM group compared with Low-dose IVM (0.6 vs. 1.2%), with an odds ratio of 0.472 (95% CI 0.209–1.067; *p* = 0.065). There were no significant differences in mortality rate (1.2 vs. 1.6% odds ratio of 0.733) (95% CI 0.392–1.369; NS).

Regarding clinical evolution of subjects with an age of ≥ 40 year old (Low-dose IVM *n* = 645 and High-dose IVM *n* = 1,016), there was a non-significant tendency toward lower ICU admission in the High-dose IVM group (0.8 vs. 1.7% with an odds ratio of 0.457) (95% CI 0.183–1.143; *p* = 0.086). There were no significant differences in mortality rate (2.1 vs. 2.9% odds ratio of 0.695) (95% CI 0.371–1.304; NS).

In the analysis of all subjects receiving High-dose IVM (*n* = 1,738) vs. C (*n* = 17,966), ICU admission was significantly lower in the High-dose IVM group compared with C (0.6 vs. 1.2%), with an odds ratio of 0.494 (95% CI 0.261–0.934, NNT 172; *p* = 0.027) and mortality rate was lower in the High-dose IVM group compared with C (1.2 vs. 2.1% odds ratio of 0.566) (95% CI 0.364–0.881, NNT 111; *p* = 0.01). Similarly, simple logistic regression analysis of ICU admission and mortality rate in High-dose IVM, Low-dose IVM and C showed that meanwhile there were no significant differences in ICU admission (exponential *B* 1.046, 95% CI 0.607–1.802; NS) or mortality rate (exponential *B* 0.773, 95% CI 0.486–1.230; NS) between Low-dose IVM compared to C, High-dose IVM was associated with lower ICU admission (exponential *B* 0.494, 95% CI 0.261–0.934; *p* = 0.03) and mortality rate (exponential *B* 0.566, 95% CI 0.364–0.881; *p* = 0.012) compared with C. No significant differences were observed between groups regarding ICU admission and mortality rate in subjects younger than 40 year old (data not shown).

Regarding clinical evolution of subjects with an age of ≥ 40 year old (C *n* = 9,212; IVM-ITT *n* = 1,016), ICU admission was significantly lower in the High-dose IVM group compared with C (0.8 vs. 2.0%), with an odds ratio of 0.383 (95% CI 0.188–0.780, NNT 81; *p* = 0.006). Similarly, mortality rate was lower in the High-dose IVM group compared with C (2.1 vs. 4.1% odds ratio of 0.498) (95% CI 0.319–0.776, NNT 50; *p* = 0.002). Simple logistic regression analysis of ICU admission and mortality rate in High-dose IVM, Low-dose IVM and C showed that meanwhile there were no significant differences in ICU admission (exponential *B* 0.838, 95% CI 0.454–1.548; NS) or mortality rate (exponential *B* 0.716, 95% CI 0.448–1.143; NS) between Low-dose IVM compared to C, High-dose IVM was associated with lower ICU admission (exponential *B* 0.383, 95% CI 0.188–0.780; *p* = 0.008) and mortality rate (exponential *B* 0.498, 95% CI 0.319–0.776; *p* = 0.002) compared with C.

**Figure 3:**
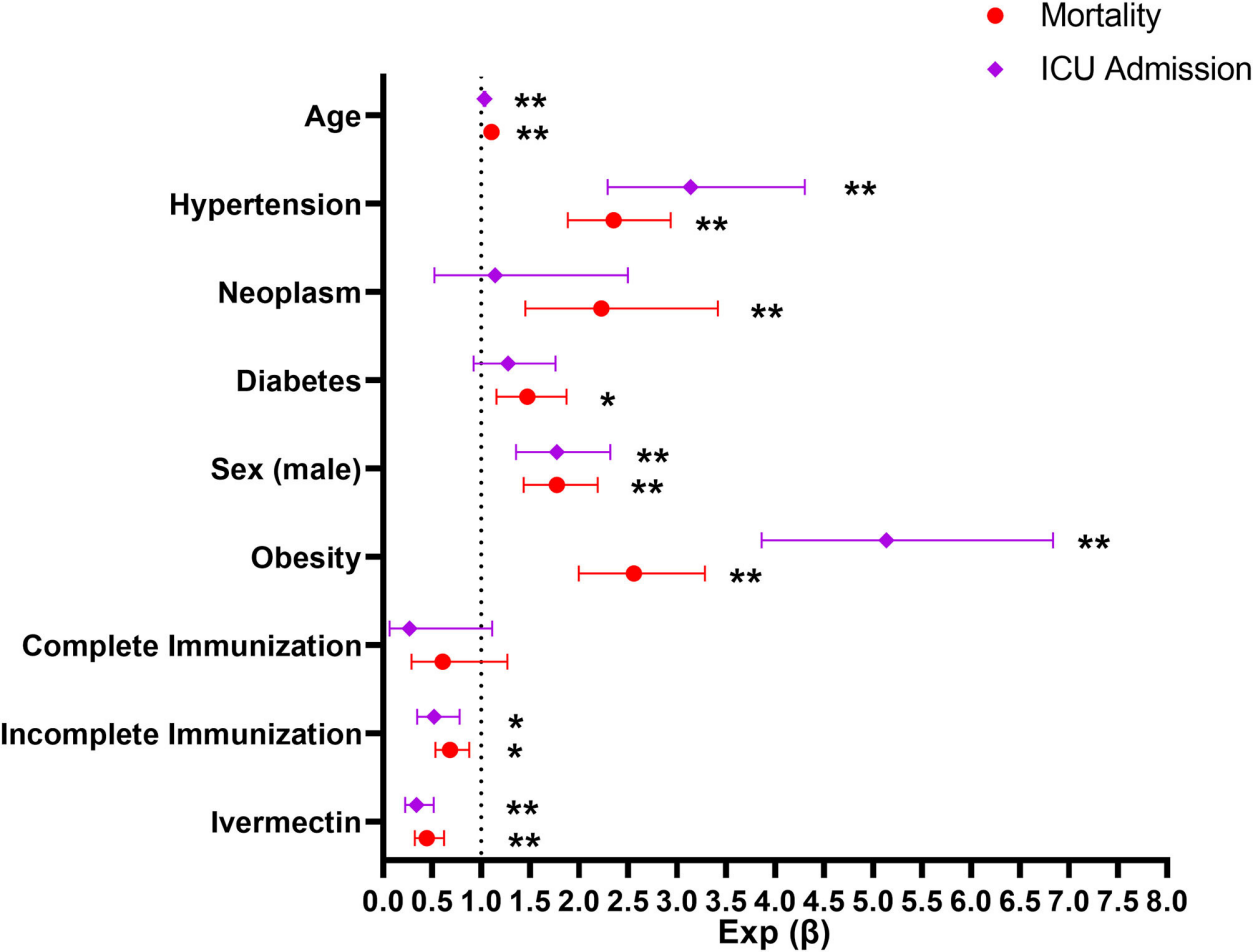
ICU admission and mortality in patients receiving ivermectin within MEURI program vs. Control after controlling for other variables by multiple logistic regression analysis. Age was considered as a continuous variable. Subjects were considered immunized after 14 days of their last vaccine dose. **p < 0.001; *p < 0.01.

A logistic regression analysis was performed after adjusting for sex, age, immunization status and comorbidities, and IVM treatment remained negatively associated with ICU admission rate and mortality rate (Figure 3).

## Discussion

This report of a monitored intervention program with ivermectin in COVID-19 patients provides observational data on a significant number of adult patients that through the incorporation of clinical and demographic data from a large number of patients from the same province and period but not participating in the program (control group) allowed a comparison and analysis of hard clinical endpoints as are admission to ICU and death. This comparison provides results that suggest a significant positive clinical impact of this intervention that, in the context of a lack of proven antiviral alternative treatments for ambulatory patients against COVID-19, the safety, availability and affordability of IVM and the growing concerns on vaccine efficacy against emerging variants of SARS-CoV-2, deserves consideration as a potential tool for case management.

The approach taken in the Province of La Pampa for the use of IVM through a MEURI Program supported and leaded by the provincial Ministry of Health was based on preliminary but inconclusive data on efficacy and a more solid reference base on the safety of the drug, even at higher doses than those approved for other indications in Argentina, as strongyloidiasis and scabies (23). The selection of high-doses of IVM was based on the results of the *in-vitro* reports on the antiviral activity of IVM against SARS-CoV-2 as well as the growing body of evidence on the safety of these higher than usual doses for other indications in pediatric and adult populations (23–25). With the incorporation of over three thousand cases that completed treatment and follow up, including 1022 with clinical laboratory monitoring, this intervention program contributes the largest analysis on the safety of high dose ivermectin at a regimen of 600 μg/kg/day for 5 consecutive days. This regimen was selected based on a proof-of-concept trial that identified a significant antiviral activity against SARS-CoV-2 in a subgroup of participant who achieved high IVM median plasma concentrations without any significant safety issues (17). In view of that seemingly dose response antiviral effect, participants of our program were advised to ingest the daily doses of IVM after a meal with adequate fat content in view of the lipophilic nature of IVM (13, 26). The favorable safety profile of this regimen of high dose IVM, which includes the identification of adverse events that were comparable in type and severity to those reported in a meta-analysis on the safety of high-dose IVM (23), in a setting with conditions for high oral bioavailability, confirms prior communications with smaller sample sizes, on the safety of these regimens and allows to focus in exploring the clinical efficacy of these regimens for a variety of clinical indications for which IVM is in pre-clinical and clinical development as a repurposed drug including COVID-19, dengue, trichuriasis and malaria. The finding of a single case of clinically significant increase in liver enzymes, in an individual with baseline abnormal values highlights these clinical and laboratory findings and provides evidence for the design of simplified MEURI Programs, should they be considered appropriate.

The controversial findings around the efficacy of IVM in COVID-19 is currently preventing from making firm recommendations to clinicians. The situation is worsened by confusing messages through social and traditional media plus articles from peer-reviewed journals that are published and afterwards retracted as well as the uncontrolled use of medical and veterinary products by the population (27). Meta-analyses that included studies with a variety of regimens have reached different interpretations and conclusions, preventing the achievement of consistent findings (14, 18, 28, 29). In this context, a MEURI Program appears as the mean to attempt monitored and controlled use of a treatment not approved for the indication but with preliminary results of adequate safety and potential efficacy. While the impossibility to perform clinical trials was not an absolute situation at the time of the design of our Program, the means and capacities of a provincial Ministry of Health were beyond the scope and resources available.

The role of IVM against SARS-CoV-2 was supported by the biologic plausibility based on *in vitro* and *in vivo* studies of mechanistic analyses and antiviral effects. Its proposed antiviral mechanism is thought to be mediated by its ability to inhibit the nuclear import of viral proteins mediated by IMPa/b1 heterodimer, and the promotion of defense mechanisms such as pyroptosis in infected epithelial cells, suggesting its possible role as a broad spectrum antiviral agent (30, 31). Although an immunomodulatory effect of IVM has been proposed by other authors (32), these effects might explain the antiviral activity against SARS-CoV-2 reported by our group and Biber et al., in two small randomized controlled trials (17, 19). However, randomized clinical trials published on this topic have shown a lack of efficacy regarding clinical outcomes. Lopez Medina et al. reported on the failure to show a significant effect of 300 μg/kg of IVM for 5 consecutive days vs. placebo in symptom's resolution among 400 patients with mild disease recruited during the first week of COVID-19 in a single center in Colombia (15). This trial, that was originally designed to demonstrate improvement of 2 points in WHO Ordinary Scale but suffered from fewer than estimated events, included a population with a median age of 37 year-old and administered IVM on an empty stomach, as indicated by the manufacturer, which probably prevented from maximizing oral biovailability of this highly lipophilic drug. In the study from Vallejos et al. in Argentina (16), 501 patients with mild early COVID-19 infection were randomized to receive placebo or IVM for 2 consecutive days at up to 200 μg/kg (the currently approved dose for other indications). Neither hospitalization (primary outcome) nor other secondary outcomes including polymerase chain reaction test negativity and safety outcomes showed statistically significant differences between groups in this population with fewer events than estimated a priori and a mean age of 42 year-old (SD ± 15.5).

The seemingly contrasting efficacy results between our analysis of an intervention program vs. the double-blind, placebo-controlled RCTs might not be discordant at closer look with reasons laying in several factors pertaining to IVM regimens, population size and outcomes; but potential bias in our results is another possibility to be considered and explored. The IVM regimen used in our program provided a higher total exposure to the drug through a higher dose per day, longer treatment (compared to the trial by Vallejos) and administration with food, which while equally safe might have allowed reaching drug levels at the relevant tissues above the threshold required for an antiviral activity resulting in better clinical outcomes (16). In terms of the populations included in the analyses, it is relevant to consider that both RCTs reached fewer primary endpoints than estimated for the sample size calculation; notwithstanding those potential limitations, which might have been affected by the age of the population recruited to those trials, neither adjustments nor secondary outcomes identified any significant clinical findings. Despite sound trial methodology, failure to demonstrate the effect of an intervention might reside in contextual elements as is the recruitment of subjects at very low risk of achieving the primary outcome regardless of the use of an intervention (33, 34), as might have been the case in both RCTs as well as our observational analysis, which due to the significantly larger population size, was able to identify a statistically significant treatment effect in admission to ICU and death in restricting the analysis to those >40 year-old.

Statistically significant differences in observational studies should be viewed with caution since the clinical and public health relevance of those results might not be judged as relevant despite the statistics. Based on that, NNT ratios provide an indicator that could inform clinicians and policy makers on the value and convenience of this intervention. Effect size is another element to be considered in the evaluation of an intervention, since the ability of trials to rule-out the effect of an intervention grows in the required sample size in direct relationship to the decrease in the effect size, with direct implications in the feasibility of a clinical trial and the convenience of an intervention (35). In the present analysis, as expected, NNT related to ICU admission and mortality prevention differed significantly between subgroups, with a lower NNT in higher risk groups, as subjects older than 40 year-old.

As an observational intervention, our analyses are susceptible to bias, which constitute the most significant limitation of this report and a major concern in COVID-19 in view of the multitude of publications of studies and observations designed, run and published at unprecedented speed (36). The risk of confounding factors introducing bias in the comparison between groups cannot be completely ruled out, although several measures were taken to minimize its occurrence, like the verification of balanced age distribution, vaccination status and prevalence of comorbidities, with a special attention paid to current oncologic processes that could identify patients with terminal disease. In reference to it, in a sub-analysis that excluded individuals with current neoplams the differences between IVM and C groups remained significant. Survivor bias was assessed and controlled in a sub-analysis (data not shown) through the exclusion from the analysis of all individuals in the control groups whose death occurred within the first 4 days since diagnosis, since those individuals were in all likelihood not offered the intervention, maintaining a significant association in favor of IVM in terms of mortality frequency in the higher risk groups (non-immunized subjects older than 40 year-old). In order to limit the mortality assessment to death related to COVID-19 in the C group, deaths were only considered for the analysis when occurring during the original hospital admission or within a month after discharge. Regarding the possible influence of differences in the prevalence of comorbidities between groups, it is important to highlight that, meanwhile most comorbidities were balanced between groups, a higher percentage of participants in the IVM group reported hypertension and obesity compared to C. Although these differences could be attributed to methodological differences in the identification of comorbidities, should they be real, describe a higher risk of disease progression and, consequently, would not explain the better outcomes observed in this group.

A per-protocol analysis including only individuals that completed the whole treatment, which could provide information of the full potential of the regimen was not performed since individuals on therapy that had their treatments interrupted at hospital admission were identified and given that situation, performing a per-protocol analysis would have given biased results that would wrongfully inflate the efficacy of the intervention.

One limitation of the present work is the absence of a detailed record of the refusal of patients to participate in the IVM program. Specifically, since the start of the program in January 2021, every physician in the province was authorized to offer this treatment to patients who met the inclusion criteria. However, the inclusion of patients was left to the discretion of the treating physician and to the acceptance by the patient. For this reason, the non-inclusion of patients in the IVM program could be due both to the refusal of patients to receive treatment after it was offered, to non-compliance with the inclusion criteria, or to the decision by the treating physician not to offer this treatment option. Unfortunately, no information is available to confirm the reason for not including each individual patient. Another limitation is the lack of pharmacokinetic data to identify a relationship between drug levels and clinical findings, which was beyond the scope of the Program.

When looking at our findings in the context of the results and conclusions of rigorous RCTs, other observational clinical studies, virologic and molecular biology studies for the evaluation of IVM in COVID-19, we conclude on the plausibility and potential clinical utility of IVM at higher doses than currently approved, optimizing its bioavailability but with a relatively moderate effect size in high-risk population groups. Without safety concerns at the doses used in our program, this report highlights IVM as an intervention that deserves a dispassionate, careful, public health-based consideration for the treatment of patients during present COVID-19 pandemic until superior therapeutic alternatives become available and affordable, and highlights the importance of performing adequately powered RCTs in order to confirm our findings. Comparisons between repurposed molecules like IVM vs. newly developed drugs like molnupiravir, tixegevimab-cilgavimab or remdesivir in terms of safety, efficacy, cost and availability will help identifying the potential role of each of these commercially available drugs for the management of COVID-19 patients.

## Data Availability Statement

The raw data supporting the conclusions of this article will be made available by the authors, without undue reservation.

## Ethics Statement

The studies involving human participants were reviewed and approved by the Provincial Ethics Committee of La Pampa, and participating individuals provided written informed consent. The patients/participants provided their written informed consent to participate in this study.

## Author Contributions

AF, MB, AP, JB, CD, MM, MAM, AK, GV, and MK: conceptualization. AF, MB, CE, AP, JB, CD, MM, and JO: data curation. MAM, AK, PF, AF, and MK: formal analysis. MAM, AK, AF, MB, JB, MM, AP, CD, CE, AB, GV, and MK: investigation. AF, MB, JB, MM, AP, CD, and MK: methodology. MAM, GV, and MK: supervisión. MAM, AK, and MK: writing—original draft. MAM, AK, AF, MB, JB, MM, AP, CD, CE, AB, PF, GV, and MK: writing—review and editing. All authors contributed to the article and approved the submitted version.

## Funding

This work was supported by the Government of La Pampa, Argentina.

## Conflict of Interest

The authors declare that the research was conducted in the absence of any commercial or financial relationships that could be construed as a potential conflict of interest.

## Publisher's Note

All claims expressed in this article are solely those of the authors and do not necessarily represent those of their affiliated organizations, or those of the publisher, the editors and the reviewers. Any product that may be evaluated in this article, or claim that may be made by its manufacturer, is not guaranteed or endorsed by the publisher.
